# SUPPORTING FAMILY CAREGIVERS: HOW DOES RELATIONSHIP STRAIN OCCUR IN CAREGIVING DYADS? A QUALITATIVE STUDY

**DOI:** 10.1093/geroni/igz038.1066

**Published:** 2019-11-08

**Authors:** Elizabeth S Avent, Laura Rath, Kylie Meyer, Donna Benton, Paul Nash

**Affiliations:** 1 University of Southern California, Los Angeles, California, United States; 2 Archstone Foundation, Long Beach, California, United States; 3 University of Texas Health Science Center San Antonio, San Antonio, Texas, United States; 4 University of Southern California, LA, California, United States

## Abstract

Family members and spouses are usually the primary caregivers for older adults. Providing direct care can be stressful, strenuous, and time-consuming for caregivers, potentially leading to frustration and anger towards care recipients. This can be detrimental to the relationship quality of the caregiving dyad. Though caregiver strain and burden have been extensively studied, there is limited information on the development of relationship strain. To explore how relationship strain occurs between caregivers and care recipients, 8 focus groups (N=62) and 8 semi-structured telephone interviews were conducted with caregivers in Los Angeles, inquiring about relationship quality with their care recipients and when frustration and anger occurs. Inductive coding was used to create coding schemas. Findings showed that most caregivers reported relationship strain occurring after taking on the caregiving role, and frustration and anger arose when providing ADLs, especially during bathing and toileting. Although these caregivers had initially experienced strain in their relationships, a recurring theme that emerged was that they developed strategies to decrease frustration and anger and improve the quality of their relationships with their care recipients. Direct communication with caregivers is important in designing a structured and effective intervention. These findings help inform an intervention for new caregivers to help them identify what can lead to relationship strain, as well as teach them reliable strategies to manage frustration and anger towards their care recipients.

